# Palestinian law to protect family and prevent violence: challenges with public opinion

**DOI:** 10.1186/s12889-023-15276-9

**Published:** 2023-03-01

**Authors:** Fayez Mahamid, Muayad Hattab, Denise Berte

**Affiliations:** 1 An-Najah National University, Nablus, Palestine; 2 Peaceful Families Project Inc., Nablus, Palestine; 3 Psychology and Counseling Department, An-Najah National University, An-Najah National University, Nablus, Palestine

**Keywords:** Domestic violence, Family Protection, Palestine, Gender-based violence

## Abstract

The Palestinian Family Protection Law was submitted for ratification in November 2020 after much collaboration between advocates, attorneys and governmental officials, as well as community and international organizations. The draft bill addresses a myriad of social issues affecting vulnerable populations in the West Bank of Palestine, including: the legal age for marriage; gender inequality in inheritance; divorce; gender-based violence; and domestic violence. However, immediate feedback from local religious scholars, with increasing pressure from the general Palestinian population, was deeply divided, with negative perceptions relating to the perceived ‘foreign’ nature of the regulations and criticism that the law was not in accordance with Palestinian culture and Islamic Law. This criticism led to two years of inactivity regarding the bill. The current study sought to evaluate the attitudes, behavior and beliefs regarding the underlying human rights issues and assumptions concerning gender, marriage, and domestic violence that could be found in the codices of the proposed legislation as well as among the general population of Palestine. The data demonstrates that the population sampled viewed the underlying premise of the Family Protection Bill negatively. Individuals with a graduate degree or above tended to view the bill with less negativity, whilst females viewed the bill more positively than males. The results of this study illustrate that, with regard to legislation that relates to family life in Palestine, there is a wide gap between the points of view held by human rights advocates and NGO’s and those held by the general population. To further the agenda of family protection community education relating to social issues, the fundamentals of Sharia law and national sovereignty may be needed so as to clarify the core Islamic beliefs in relation to human rights and oppression, as well as to increase Palestinian ownership of the family protection agenda.

## Introduction

In light of the prevailing culture, the great influence of Islamic scholars (*Ulama*) and the influence of patriarchy in Arab and Islamic society, violence against women remains a taboo subject in social conversation and is considered to be an internal family matter in much of the Muslim world. Few Arab/Muslim majority countries have managed to pass laws to protect women from domestic violence. In those countries that have, the legislation is limited in scale with regard to intervening in domestic affairs [12]. The countries that currently provide provisions in law regarding violence against women are: Jordan, Saudi Arabia, Lebanon, Algeria, and Bahrain. The United Arab Emirates, Yemen and Tunisia allocate specific provisions in the penal code that address the issue [22]. Most Arab/Muslim majority countries neglect to refer to gender-based violence and domestic violence (GBV), defining it as out of the scope of their legal authority [12].

In the last two years, social and legal debate has escalated in Palestine over the proposed “Family Protection Law”, a wide ranging social legislation that addresses a multitude of issues related to gender, marriage, and violence against women. The Palestinian Authority has failed to approve the law further to the protests of Palestinian communities, religious scholars and religious leaders. Simultaneously, human rights and women’s organizations are intensifying their activism and exerting pressure for the law to be passed in light of the rising rates of violence against women [38].

Amid this controversy and opposition, the Palestinian Central Bureau of Statistics reported in its official annual statistics on domestic violence [27] that 52.2% of currently or previously married women, were subjected to emotional abuse, making it the most common form of violence against women in the region. The data also showed that 27.2% of married or previously married women were subjected to physical violence from their husbands on at least one occasion. The Palestinian Central Bureau of Statistics demonstrated that there has been a decrease in the level of reported violence from 37% to 2011 to 27% in 2019. The reasons for this decrease are suspected to be associated with both the social and cultural beliefs against involving law enforcement in family matters as well as the actual obstacles that exist for women who desire to report this type of abuse. This theory is supported by statistics which show that, 60.1% of Palestinian victims of domestic violence preferred to remain silent, with only 1% going to the local police headquarters or the Family Protection Unit to file a complaint or seek assistance. [27] According to research conducted by the Palestinian Working Woman Society for Development (PWWSD) [18], social norms are the main reason that women refuse to report any form of abuse or violence against them. For example, the victim’s fear for her own safety, the fear of shame and social stigmatization, feelings of guilt and the belief that violence is a natural and expected behaviour by a husband, fear of being abandoned, and the fear of her children’s financial stability, are just some examples of why women preferred to remain silent in the face of domestic violence [18].

Against this background, the Palestinian government, and both local and international women’s organizations, joined forces to create comprehensive legislation to protect family members, especially women and children, from domestic violence, as well as address issues of gender imbalance in employment, inheritance, etc. However, their efforts have been thwarted by a variety of social and political factors that have stalled the certification of the new law.

## Historical background

The idea of ​creating a comprehensive law to protect Palestinian families from violence and women from social inequality, began in 2004 after discussions between civil society and women’s organizations, representatives of political parties, and specialists in legal and social welfare, noted a rise in domestic violence that was having a negative impact on Palestinian society [20]. Beyond the need to provide protection for women and children from domestic violence and enhance their access to justice, these discussions were also targeted at the need to combat discrimination against women that might be found in the applied laws of Palestine (such as inheritance, employment, etc.) or in social and cultural common practice [42].

In 2008, following a national conference held in December 2008 under the name, “Towards the Adoption of the Family Protection from Violence Law”, it was announced that a draft law to protect families from domestic violence would be presented for community discussion, [24]. In 2012, the Ministry of Women’s Affairs assigned the draft law to the Palestinian Council of Ministers, following which the Council included it on its agenda in 2013 and referred it to all ministries for comment. The previous Council of Ministers completed the final version of the draft Family Protection Law in June 2016, and called for national consultations to discuss the project with civil work institutions and specialists; however, the law was not issued by the Palestinian President at that time.

With the participation of civil society and women’s institutions, the draft Family Protection Law was subject to additional review and amendment by the current Palestinian government, and was approved after its first reading by the Council of Ministers on the 5th November 2020. However, this review process was met with an opposition campaign that included claims of western interference and infidelity to the religious and cultural foundations of the Palestinian context, with claims that the texts of the draft were derived from the Convention on the Elimination of All Forms of Discrimination against Women (CEDAW) that “contradicts Islamic law” [29]. In April 2014, Palestine became a signatory to the CEDAW, indicating that the Palestinian state was committed to taking all necessary means and measures to prohibit discrimination against women, as well as work to combat it. While this move by the Palestinian state was welcomed by international and human rights organizations, it has fostered internal debate between those who support the progressive liberalization of Palestinian law and who are pressuring the government to expedite it, and those who see westernization as a forced external process that will lead to the demolition of Islamic Sharia provisions [1, 40], and is an attack on Palestinian culture [11].

Accordingly, in 2020, as the draft law was leaked to the press and social media, opponents to the draft linked it to the CEDAW convention, which had been previously criticized for its anti-Islamic values and immorality [3]. Although there is no mention of the Convention in the law, a number of articles were very similar, which fueled the idea that it was derived from the CEDAW and was a representation of an external, forced change in Palestinian life.

While the vast majority of Muslim religious scholars agree on issues such as a woman’s right to education and work, their views and opinions of women and their role within society begin to diverge, depending on their education and cultural influences. For example, not all local Palestinian scholars accept that women have equal rights in complex areas such as marriage, divorce, inheritance, and a woman’s right to refuse sexual relations with their spouse [5, 37]. Furthermore, most Muslim scholars in Palestine discourage any changes to the Personal Status Law that would give equal rights to women in these aforementioned areas [5, 29]. There are also solid objections to the passing of any legal code or article that relates to marital rape, or that would deny the guardianship of men over women in general, or the guardianship of a husband over his wife and children in particular [35]. However, it does not follow that all Muslim scholars accept these traditional views regarding women. There are other, enlightened scholars who have argued that Islam should be interpreted in compliance with a modern understanding of human rights and gender equality. For example, a lecturer in Islamic law and a member of the Association of Muslim Scholars pointed out that the draft law does not contradict the texts of Islamic law [2, 23]. This scholar stated that the Personal Status Law is based on jurisprudential interpretations that are not agreed upon among scholars (*ulama*), meaning that it is subject to change and development in order to meet the modern needs of society [26, 23].

The Palestinian Authority appeared to pause the promotion of the bill, appearing not to know how to address the cultural and religious objections to the law. On the other hand, on the 6th August 2020, the Independent Commission for Human Rights in Palestine issued a statement condemning the accusations of blasphemy and threats against those agreeing with the bill, given that the law was drafted in response to a national demand and societal need to confront the increasing domestic violence and murder of women [20].

Palestinian human rights advocates and national political parties did not appear to take the conflict seriously, failing to respond to the attack on women’s organizations, not responding to the campaigns of threats and accusations of infidelity against the support of the draft law, and demonstrating little interest in the social agenda and gender equality. Conservative/Islamic populous movements, however, were able to mobilize support to prevent any attempt by the government to amend the legal ruling or jurisprudence in Palestine that could provide protection for women from domestic or cultural violence to which they are exposed [7].

While the politicians reviewed the law, official data published in 2019 presented alarming patterns in relation to violence against women and children in Palestine [27]. The number of reported murders of women in the West Bank and Gaza Strip reached over 140 between the years 2014–2019. The number of children (12–17 years) who were exposed to physical violence from their father was 26%, with emotional abuse at 63%; this compared to 24.8% who were subjected to physical violence from their mother with 62% experiencing emotional abuse. Furthermore, despite the limited decrease in the level of violence against women between the years 2011–2019, the percentage of women who have been exposed to domestic violence remained high. The data showed that approximately 29% of married or previously married women were subjected to a form of violence from their husband on at least one occasion. This data has been used by the Ministry of Social Development, women’s social organizations, human rights groups and activists, to call for an expeditious implementation of the Family Protection Law, which has been a consistent demand since 2006 [24].

## The proposed draft family protection law

The proposed Family Protection Law (FPL) contains 52 articles, and was passed after its first reading at the Council of Ministers on the 5th November 2020. The law must pass three readings before it can be sent to the president for approval. Once approved by the president as a decree and published in the Palestine Gazette, it will become law, effective 28 days from the date of its publication [41].

## Definitions & scope of protection

Article 1 provides definitions of the most important terms used in the FPL, most notably the definition of family violence. The draft law defines ‘family violence’ as, “any act or failure to act by a family member against another family member that causes physical, or psychological harm, including exploitation, physical, psychological and sexual mistreatment, and forced labor, or the threat of these acts, whether the act or threat occurred within the family home or outside it.”

The draft has also provided a specific definition to the term, ‘economic violence and forced labor’, as, a “prohibition from work, or forcing labor, or controlling its returns and remuneration, and includes any control over property and inheritance rights, concealment of money, control over any movable or immovable property shared, and/or prohibition from using or disposing of it”.

According to official Palestinian data, 37% of married or previously married women were subjected to economic violence, which makes this category the second most common type of violence against women in Palestine. The data includes issues relating to inheritance whereby many women, when demanding their right of possession in shared inheritance, face obstacles including; intimidation, psychological abuse, and physical violence. On some occasions, this leads to the homicide of women [4] who have insisted on claiming their property and inheritance rights, by their brothers or other relatives, these crimes being justified on the grounds of protecting family ‘honor’ [30], a term that has been socially and legally assimilated as a supreme value in patriarchal societies, including Palestine [42]. In addition to the cultural obstacles women face in Palestine, access to the justice system and the complex procedures involved in the Palestinian courts, including expensive legal costs, leave women without legal advice and hesitant to pursue any independent legal claim before the courts [6].

As to the scope of protection, Article (4) defines ‘protected family members’ as: those who have a blood or kinship relationship up to the third degree, such as a husband and wife; individuals who are related by blood up to the fourth degree, provided that they live in one dwelling; former spouses, provided that they have joint children; employees in homes; and any party linked to the family through adoption (for Christians) or surrogate families and foster care (for Muslims) [19, 33].

## Aim & purpose of the law

Article 3 of the draft states that the aim and purpose of the law is to preserve family harmony and the unity of Palestinian society. The FPL aims to protect family members from all forms of violence, ensuring that victims of domestic violence have access to justice with the best interests of the victim in mind, providing victims of domestic violence with psychological, physical and economical rehabilitation, as well as reintegration into society, if needed. The final aim of the FPL is to ensure accountability, punishment, and the rehabilitation of perpetrators of domestic violence as well as their reintegration into society [35].

To achieve these aims, the FPL provides, in its subsequent articles, methods and procedures for implementation. Accordingly, the Ministry of Social Development must (under Articles 5–7), create relevant centers to educate the public against domestic violence, provide rehabilitation and treatment to both victims and aggressors, and provide shelter and health assistance to victims and their children, as well as other services that are necessary to implement the law [19, 33].

## Family protection counsellors & mediation

Under Articles 7–11 of the FPL, the Ministry of Social Development must appoint Family Protection Counsellors (FPCs) whose main duty is to implement the law, and who will, accordingly, have judicial enforcement rights as bailiffs. The FPC will have other duties including: conducting interviews or investigations with individuals and families to assess potential domestic violence situations; maintaining accurate records and preparing reports for legal action; giving evidence in court; offering information and support to the public, families, and victims; providing mediation, when possible, to solve family disputes; and acting or proposing necessary remedies to prevent any threat of assault against a family member [19, 31]. Article (14) provides an alternative course of action by way of family mediation. Mediation can be permitted in special circumstances, and on only one occasion, if an assault does not involve any accusation or allegation of sexual harassment or violence against a child, the elderly or a disabled person. Mediation can be attended or requested through the PFC office, or upon an order by the Family Protection Prosecution [33], whose role is explained below.

## family protection (application, prosecution, & protection order)

Article (13) states that a specialized Family Protection Prosecution (FPP) department, as a branch of the Public Prosecution department, must be formed in order to: follow and manage all matters relating to family violence and family protection from violence; proceed with full investigations of any alleged crimes of domestic violence; and take any necessary measures to protect a family and its members from an aggressor [34].

Article (15) allows a member of the family, or any member of the public, to report incidents of family violence to the relevant authority. Article (17) provides full protection to any person who reports, to an appropriate authority, a crime of domestic violence, or who files a complaint with the police against any family member for family violence. Such protection includes; keeping the identity and name of the witness or accuser anonymous, and giving them a full amnesty from prosecution due to them making a domestic violence report or submitting a complaint of family violence [25].

Article (16) makes it compulsory for anyone who works in the health or education sector, social services, or legal services, whether in the public or private sector, to report to the FPS or an appropriate agency, “upon her or his knowledge, by virtue of his work, of the occurrence of any of the crimes of domestic violence specified in this law”. A person who works in any of the above sectors can be sentenced to 3–12 months’ imprisonment for withholding such knowledge or refusing to report it to the appropriate authority [10].

The FPL has introduced, for the first time in the Palestinian legal system, the right to issue a Protection Order. Articles (18)–(24) provide when and how such an order can be issued by either the FPP or the court. An application for a Protection Order can be requested from the FPP by: the victim; the victim’s representative; a family member of the victim; the sponsor of the victim; any witnesses; or the guardian or warden of the victim. In the event that the guardian or custodian is the perpetrator of the domestic violence, protection can be requested from a family member up to the third degree [32].

Article (25) also states that, even before trial, a Protection Order may be issued based on domestic violence and can include “removing the abuser from the home for a temporary period determined by the jurisdiction.“ Articles (26) and (27) gives a judge the right to extend or suspend the Protection Order, whilst Article (28) provides the punishments that can be handed down to an aggressor. These include a fine and imprisonment for up to 12 months if the aggressor contravenes the terms of the Protection Order [13, 20].

## Jurisprudence & criminalization

Articles (29)–(38) establish the rules of investigation and the applicable legal regulations and court procedures that must be followed in court trials involving domestic violence, including the victim’s right to confidentiality [28]. Article (39), in its second paragraph, gives the court the right to remove guardianship from an aggressor who is accused of domestic violence, even if the aggressor is the father, husband or sponsor of the victim. Opponents argued that the removal of a father’s guardianship in relation to his child is harsh and contradicts Islamic rules that give a father a full right of guardianship, except in limited and extreme situations, such as insanity or infidelity [8].

Article (40) states that economic violence against a member of a family is prohibited. According to the definition contained in the draft, this may include actions such as prohibiting a family member from working. This article has been criticized by opponents as having a wide definition of economic violence and being in contradiction to Palestinian culture that provides for the father or guardian to have the right to determine what type of employment his children (especially his daughters), and wife can engage in.

Article (42) criminalizes, for the first time in Palestinian jurisprudence, marital rape or sexual acts carried out against the wishes of a spouse. This section was greatly criticized by Palestinian Islamic scholars and clerics who saw it as a westernized concept opposed to Islamic rules. Islamic rules (as per the interpretation of current thought) makes it clear that, while a husband has privileges regarding sexual intercourse, sexual activities should be enjoyed and respected by both parties [37]. In this interpretation, the existence of marital rape is not congruent with Islamic teaching or local thought [39].

Opponents also argued that Article (41), which deals with harassment, has too broad a definition and interferes with family life in a way that violates the purpose of the draft law. According to the draft, harassment within the family includes, “any harassment that a person inflicts on his family members verbally, by touching, or by gestures that would undermine the victim’s dignity, infringe his modesty, or address his privacy or feelings.“ These provisions are considered, by some opponents, to be exaggerated, considering that the family relationship should bear ‘a degree of flexibility’ in this regard, especially between a husband and wife, and a distinction should be made between ‘normal behavior’ and what could constitute ‘actual aggression’.

Article (43) stipulates a penalty for, “anyone who uses psychological/emotional abuse against a member of his family.“ According to the first article of the law, psychological violence includes: “swearing, terrorizations, threats, insults, intimidation, slander, or defamation.” Opponents of the law are of the view that the definition of psychological violence is too broad and contains terms that are indistinct and which can be interpreted in different ways. For example, in Arabic, the terms, ‘insult’ and ‘threat’ are vague and indefinite, and can include any sentence which, on the one hand, may be viewed by some in society as mild to normal or, on the other, as being harsh and abusive. Accordingly, under this article, a parent may be imprisoned for attempting to correct the behavior of their child if they include simple threats such as, “you will not be allowed to watch TV tonight”, or, “you will not be allowed to play out today”. Likewise, a husband or wife could be imprisoned in the event that either of them say a sentence that includes a threat or insult against the other [16].

Article (44) discusses the punishment of, “anyone who commits a crime of gender discrimination” [9, 10]. Article (1) defines discrimination as, “any distinction, exclusion or restriction practiced by a family member over other members of the family on the basis of gender and has the effect or purpose of impairing, insulting, or the denial of human rights and fundamental freedoms in the political, economic, social or cultural field”. Opponents have argued that the definition is too broad and conflicts with Islamic and cultural values that respect the role and humanity of each gender whilst not accepting full and boundless equality [21].

Finally, Article (52) provides for the abolition of, “anything that conflicts with the provisions of this Law by Decree”. This provision has been criticized by opponents for canceling, in their view, the provisions of a father’s guardianship over his children that is stipulated in the Personal Status Law, as well as canceling the articles in the Palestinian Criminal Law that criminalizes a wife’s adultery. Such claims have been rejected by the Palestinian Authority and the supporters of the law, who have commented that objections by the law’s opponents are a complete twist on the meaning of the article [14].

Muslim scholars have long declared that Islam determines the worth of men and women equally, and that Islam “established equal rights for women in spiritual, social, political, and economic realms centuries before Western societies” [36]. However, local beliefs and attitudes relating to women and their role in society in specific cultural contexts are frequently inconsistent with these established Islamic religious doctrines. While the vast majority of Muslim leaders agree on issues such as a woman’s right to education and employment, many local communities are challenged when extrapolating rights on complex issues such as; equality in marriage and divorce, equal rights regarding inheritance, and a woman’s right to refuse sexual relations with her spouse [20].

It is recognized by both supporters and opponents of the draft FPL, that vulnerable groups in Palestinian society - particularly women - are subjected to unacceptable levels of violence within the family framework, both before and after marriage, and that there is a need to take measures to eliminate these practices, or at least mitigate their severity. However, despite unanimous agreement, the implementation of such measures has faced serious obstacles. While supporters of the FPL call for the immediate implementation of the law as the only way to resolve the issues, opponents argue that educating the public with Islamic values is the best, or only, solution to the issue [34].

The most prominent current problem is the intense polarization of both the supporters and opponents of the FPL [14]. While the necessity to implement a family protection law that deals with domestic violence is largely accepted, its implementation cannot be effective unless it has a process of systematic societal discussion and passes through stages of gradation that must begin with public awareness, scientific debate and legal discussion among scholars and human rights activists. In addition, a more gradual change in legislation is required and, finally the development and building on both of the above stages [21].

If social regulation of gender equity and gender-based violence is to be embedded in legislation, there is much to be understood about the social and cultural obstacles to the acceptance of these concepts, particularly in Palestine, where the contextual lens of occupation and service-based colonialization cannot be ignored. A true and honest conversation within civil society is needed, separate to those taking place between advocates and professionals who are often looked at with suspicion, due to their close ties and alliances with western organizations. While the leading voices supporting the FPL may not feel comfortable with local resistance, it is necessary to thoroughly investigate any opposition if progress is to be made.

While legislation can foster and support social change, a deep transformation of cultural norms can only be achieved through grassroots effort that affects the general population’s definition of the issues and a genuine internal impulse for a different path. When external forces are seen to be pushing an independent agenda, efforts for social change will always be met with resistance, especially in a population that has had such little opportunity to self-govern. The current investigation is a unique opportunity to begin a real dialogue with the Palestinian people in order to, firstly, appreciate their perspective, before demanding legal and societal change.

The current study was undertaken to better understand the attitudes, beliefs and behavior of a sample of Palestinian adults relating to the proposed Family Protection Law, as well as to examine the social and demographic factors that may affect an individual’s point of view. Specifically, the current study was designed to answer the following questions: (1) what are the perceptions and attitudes of Palestinians towards the family protection law? (2) Are there significant differences in the attitudes and perceptions of Palestinians toward the family protection law due to study demographic variables (i.e. gender, academic level of education and residency).

## Methods

### Participants

The study sample was drawn from Palestinians living in the West Bank of Palestine using the convenience sampling technique, the sample consisting of 285 Palestinian adults. Participants were recruited through online methods. Out of 305 participants who responded to the study instruments, 285 questionnaires were analyzed. Twenty questionnaires were excluded from the analysis due to missing and incomplete data entry. The average age of the participants was 32.10 years (*SD* = 8.32). 19.6% of the participants had obtained a Master’s degree, and 80.4% had a Bachelor degree. 31.3% of participants were males whilst 68.4% were females. Finally, 44.7% of participants were from cities, 51.3% from villages, and 4% from internally displaced camps. The criteria for including participants in the study were: (1) those without any type of severe mental illness, (2) those living in Palestine, and (3) native Arabic speakers.

### Measures

*Personal Information Questionnaire*. The participants self-reported on the following questions: Academic level, gender, age, and place of residence. No external follow-up for verification was conducted.

*Scale of Attitudes towards Family Protection Law*. This is a 19 item self-report measure developed by the authors to test knowledge, attitudes, and behavior relating to the issues addressed in the Family Protection Law. A committee of experts in psychology reviewed the items of the scale for content validity and comprehensiveness. The researchers used a score of 80% agreement between experts for the inclusion of each item. Accordingly, the researchers modified some items of the scale and changed the interpretation for others; minor modifications were also made on the basis of feedback from committee members. A 5-point scale ranging from 1 (strongly disagree) to 5 (strongly agree) allowed participants to rate each item. The cut off score of the scale ranged from 1 to 2.33 (low), 2.34 to 3.67 (moderate), and 3.67 to 5 (high). In order to test the validity of the scale, the scale was distributed to (80) participants independent of the sample of the study (the validity sample). The scale indicated a high level of construct validity in assessing participants’ attitudes towards family protection law and correlations between items, with the total score of the scale ranging between (0.41 − 0.62). Moreover, the results of an exploratory factor analysis (EFA) indicated a stable one factor construct of our scale. Finally, Cronbach’s alpha coefficients indicated high internal consistency for the total scale (0.90).

### Procedures

The study was carried out in January 2022 and targeted Palestinian adults in the West Bank of Palestine. Before embarking on the study, the research team received approval from the An-Najah Institutional Review Board (IRB). Participants were recruited using online methods due to the aims and procedures of the study being explained online, namely the subject of the study and its purpose, including ethical issues of confidentiality and voluntary participation. Individuals who sent an email clarifying their willingness to participate in the study received the instruments of study with informed consent. Completed questionnaires were analyzed using IBM SPSS 28.

### Data analysis

Mean and standard deviations were calculated to explore participants’ attitudes and perceptions toward the FPL. Moreover, frequencies and percentages for participants’ agreement with the law were explored. Finally, a three-way ANOVA test was used to find the significance of the differences between perceptions and attitudes towards a family protection law based on the study of demographic variables, i.e. gender, academic level of education, and place of residency (urban vs. rural).

## Findings

In Table 1, we reversed coded items from 10 to 19. Participants reported high scores on items 3, 4 and 5. Moderate scores were reported on items 1, 2, 7, 8, 9, 13, 14, 15, 16 and the total score. Finally, participants reported low scores on items 6, 10, 11, 12, 17, 18 and 19.

**Table 1:** Means and standard deviations for participants’ perceptions of the Family Protection Law

Item	Statement	Mean	SD
**1**	I think that the Palestinian family protection law contradicts Islamic Sharia	2.73	1.25
**2**	I do not think that the Family Protection law does justice to Palestinian women	2.92	1.28
**3**	I believe that the family protection law is based on the principles of the CEDAW agreement	3.27	1.23
**4**	I think that the CEDAW agreement is against Islamic Sharia	3.95	1.19
**5**	I think that the family protection law is influenced by western laws	3.74	1.11
**6**	I do not think that the family protection law is applicable in Palestinian society	3.08	1.32
**7**	I think that the Family Protection Law negatively affects the values of Palestinian society	2.77	1.38
**8**	I think that the family protection law contradicts the principle of guardianship of men, which is recognized by Islamic Sharia	2.69	1.41
**9**	I think that the family protection law destroys the foundations of the Palestinian family	2.72	1.37
**10**	I believe that the Palestinian family protection law enhances community mental health	2.31	1.18
**11**	I believe that the family protection law reduces the level of gender-based violence in Palestine	2.32	1.16
**12**	I think that the family protection law is concerned with psychological and social treatment towards victims	2.36	1.15
**13**	I think that the family protection law is concerned with rehabilitating and treating offenders to reduce domestic violence in Palestine	2.60	1.16
**14**	I think that the family protection law guarantees women rights by setting an appropriate age for marriage	2.63	1.15
**15**	I think that the family protection law sheds light on the psychological and social needs of the Palestinian family	2.47	1.17
**16**	I think that the family protection law enhances the compatibility and psychological well-being of the Palestinian family	2.79	1.24
**17**	I think that there is a need to prevent a husband from taking the salary of his wife	1.81	1.05
**18**	I think that there is a need to reduce violence against women and children that is prevalent in Palestinian society	1.40	0.721
**19**	I think that depriving a girl of inheritance is a problem that exists in Palestinian society and must be addressed	1.45	0.80
	**Total**	2.62	0.77

**Fig. 1:**
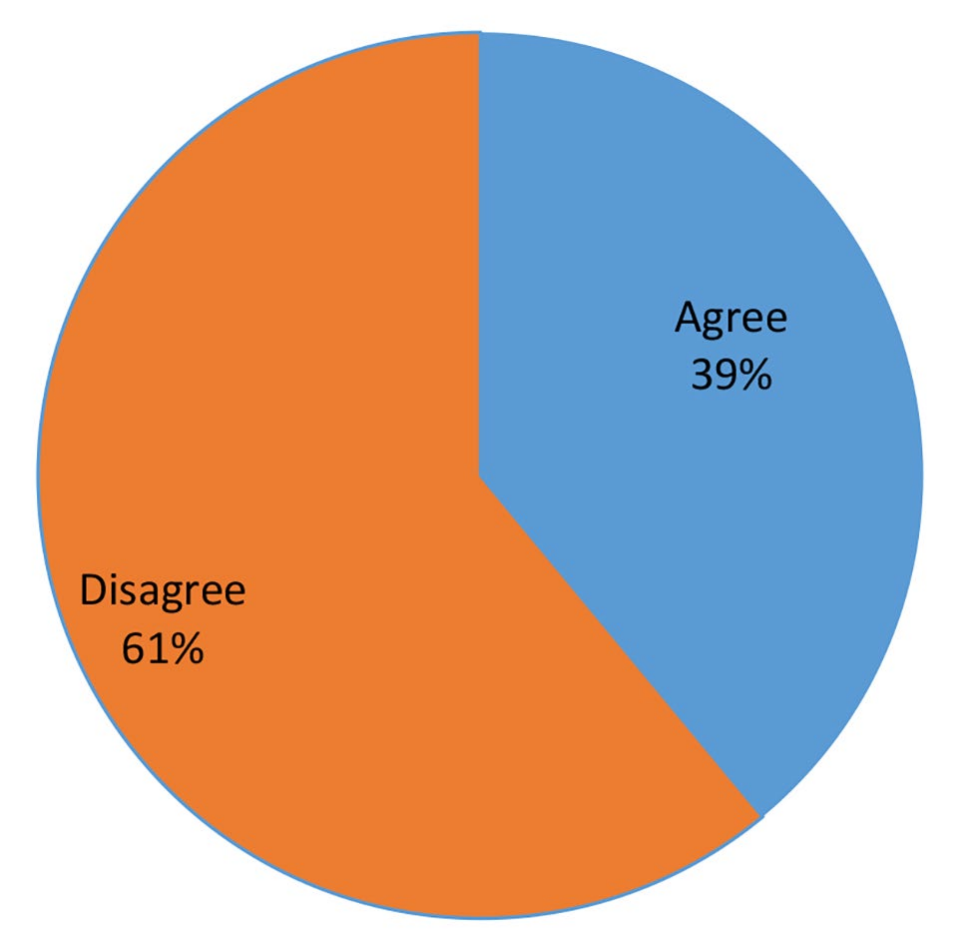
Participants’ agreement with family protection law

When participants were asked if they agreed or disagreed with the law, 39% said they agreed with the general tenets of the law, whilst 61% said they disagreed (as shown in Fig. 1).

The results in Table 2 show the differences between study variables. In order to test the significance of these differences, the three-way ANOVA test was calculated (as shown in Table 3).

**Table 2:** Means and standard deviations for study variables

Variable		Mean	SD
*Gender*	Female	2.94	0.80
	Male	2.47	0.71
*Academic level*	MA	2.98	0.78
	BA	2.53	0.75
*Residency*	Village	2.58	0.83
	City	2.64	0.72
	Internally displaced camps	2.61	0.68

**Table 3:** Three-way ANOVA to test the differences between study variables

Source	SS	df	MS	F	Sig	
*Academic*	4.26	2	2.13	3.90	**0.02	
*Gender*	7.99	1	7.99	14.60	**0.000	
*Place*	1.07	2	0.53	0.98	0.37	
*Error*	152.66	279	0.54			
*Corrected Total*	171.35	284				

a. R Squared = 0.109 (Adjusted R Squared = 0.093)

The results in Table 3 showed significant differences in the participants’ perspectives toward the Palestinian family protection law, with women reporting more positive attitudes towards the law than men. Moreover, there were significant differences found in the perspectives of participants who held BA’s and MA’s, with the more highly educated participants reporting greater levels of agreement with the law.

## Discussion

The current study showed that Palestinian adults in general have negative attitudes toward the Family Protection Law. As suspected, gender was a significant factor to be considered, with females viewing the law more positively than males. Education at the level of a master’s degree or above also produced a more favorable view regarding the law. Place of residence (rural vs. urban) did not affect the results of the survey.

The general findings of the survey may be disheartening to family law advocates who have spent several years constructing the foundation for a more just legal system in Palestine. The criticism appears to stem from two specific concerns relating to the unique situation of Palestine as an occupied territory with a weak legislative tradition. One set of concerns relates to the legislation of the home life of Palestinians and the criminalization of acts between family members. The perception that a government could or should regulate family behavior is negatively viewed by the Palestinian population who have been continually victimized (both legislatively and by enforcement agents), not only by occupation forces but also by local authorities. In a geographic area where there has been little experience of a deep governmental structure resulting in social justice initiatives, the concept of adding any institutional restriction to domestic roles and activities is frightening to the local population. Critics have suggested that charges of violation of the Family Protection Law could be raised against political leaders to silence and distract them, as has occurred with other civil and even criminal charges. Such criticism is not based on the specifics of the law but on the opening of a new set of rules that can be utilized to manipulate the population, especially in an area (the regulation of domestic roles) that is considered the sacred right of the family themselves [18].

The second area of criticism is based on the sensitivity of Palestinians regarding the sovereignty of their nation. As a people under occupation, Palestinians have very little control over anything in their territory. National boundaries, defense, elections, the justice system, natural resources, commerce, etc. are under continual negotiation with the occupying entity, with little weight given to the desires or needs of the population. In the area of social services and mental health, the agenda for the country is set by international NGO’s who are the donors and providers of most social and mental health services in the West Bank. It has long been noted that service provision is based on an underlying ‘western’ value system that focuses on trauma, gender-based inequalities and frequently pits sections of the population against each other instead of addressing the issues directly related to occupation. Frequently, in retaliation, the general population react by endorsing more conservative Islamic perspectives that are used to polarize the community and which are not representative of the daily functioning or general beliefs of the citizens at large.

While feelings about injustice against vulnerable people (children, women, widows) are generally strong, the idea of legislating rather than individual, family or community intervention being relied upon, is a major obstacle to the law. The lack of agreement regarding the last three items of the scale that discusses family violence, demonstrates this dilemma. There are subgroups within the population that are more ‘traditional’ and support the ‘right’ of physical discipline, as well as a more rigid family/social hierarchy when compared with those that exist in most other societies. However, in Palestine it is difficult to separate the desire to confront family violence with the lack of confidence in any legislative system.

In addition, the Family Protection Law appears to have an overly zealous range of proposed legal changes including: an entire system of Protective Orders; the designation of marital rape; early marriage; the criminalization of both domestic violence and child abuse; and both employment and inheritance discrimination [15]. The wide scope of the law, without cultural or structural systems for implementation, may give the feeling of a social ‘take-over’ that introduces ideals that evolved in the United States and Europe over centuries, but which are less relevant to the daily life of people living with extreme injustice under occupation.

The limitations of the current study are multiple. The sample was self-selected, and all measures were self-reported. Participants did not have an orientation to the Family Protection Law and may have been influenced by the controversy seen in social media. Many more women than men completed the survey, and little is known about their employment history, family role and/or economic status.

There has been little study of the role that religion and law has in Muslim majority countries, especially regarding Family Law issues. Sharia is, in fact, not a system of standardized law, but a religious ideal based on the sacred texts of Islam (the Quran and Hadith) thought to only be understood by the divine Allah [17]. There are 50 Muslim majority countries, all of whom discuss Sharia in their jurisprudence but share few similarities in their penal codes which are based on cultural interpretations and customs. In Saudi Arabia and Iran for example, women are legally required to cover themselves and travel with male companions. In the UAE and Qatar, none of these restrictions exist. In the Quran, it is explicitly stated that men and women are morally and religiously equal. In addition, inherent in Islamic practice is a methodology to ensure that the rights of women and children are protected (including having a woman voluntarily sign a marriage agreement, ensuring a woman’s right to divorce, protecting the right to abortion in the case of risk to a mother, and many other examples). However, the narrative from western media sources is that, when Sharia is used, the rights of women are diminished. This perception is still strong and has little neutral data to build upon [43].

A study in the summer of 2021 [21], found that a sample of mental health and social service workers had a significant, positive reaction further to undertaking training under a curriculum that defined domestic violence as against Islamic values and sacred texts (the Quran and Hadith) and which focused on family violence as a form of oppression and violation of human rights. The curriculum, created by the Peaceful Families Project, a national US based domestic violence research and resource development agency, uses Islamic materials to define and offer alternative models from an Islamic perspective. The curriculum was easily learned and accepted by participants, whilst also assisting them in increasing their motivation and resources for addressing domestic violence in their communities. It is hoped that presentations and curriculums that present domestic violence through an Islamic lens will help to bridge the gap in the Family Protection Law dilemma.

Further research and training that includes relevant stakeholders, such as: religious leaders; medical and mental health professionals; community representatives; and legislative representatives, would be greatly beneficial in getting a clearer and deeper understanding of the issues. Separating the issues and focusing on the concerns regarding each individually, along with public health/opinion campaigns, would assist policy makers in ascertaining what is needed to create a strong base of support for the legislation of domestic matters in areas of highest risk. Small legislative changes, alongside subsequent studies of impact, may create an environment of acceptance and confidence that is needed to move forward with comprehensive laws.

While it is clear that Palestinian society would benefit from a wide variety of social reforms, it is unlikely that all of them could be placed in one law without first breaking down each area, educating the community and creating social structures that would support the social changes. Curriculums, such as that introduced by the Peaceful Families Project, can assist in creating ownership and de-westernizing proposed changes. A collaboration between mental health services, social services and the legal system in Palestine would be best equipped to move forward the agenda of social reform, if not the Family Protection Bill itself.

## Data Availability

The datasets generated during and/or analyzed during the current study are available from the corresponding author on reasonable request.
